# Improving Neural Network Detection Accuracy of Electric Power Bushings in Infrared Images by Hough Transform

**DOI:** 10.3390/s20102931

**Published:** 2020-05-21

**Authors:** Hongshan Zhao, Zeyan Zhang

**Affiliations:** School of Electrical & Electronic Engineering, North China Electric Power University, Baoding 071003, China; 2182213092@ncepu.edu.cn

**Keywords:** infrared image, power apparatus bushing, standard hough transform, target detection, YOLOv2, CNN

## Abstract

To improve the neural network detection accuracy of the electric power bushings in infrared images, a modified algorithm based on the You Only Look Once version 2 (YOLOv2) network is proposed to achieve better recognition results. Specifically, YOLOv2 corresponds to a convolutional neural network (CNN), although its rotation invariance is poor, and some bounding boxes (BBs) exhibit certain deviations. To solve this problem, the standard Hough transform and image rotation are utilized to determine the optimal recognition angle for target detection, such that an optimal recognition effect of YOLOv2 on inclined objects (for example, bushing) is achieved. With respect to the problem that the BB is biased, the shape feature of the bushing is extracted by the Gap statistic algorithm, based on K-means clustering; thereafter, the sliding window (SW) is utilized to determine the optimal recognition area. Experimental verification indicates that the proposed rotating image method can improve the recognition effect, and the SW can further modify the BB. The accuracy of target detection increases to 97.33%, and the recall increases to 95%.

## 1. Introduction

When electrical equipment fails, information on certain non-electrical quantity typically contains fault information such as gas ingredients in transformer insulating oil and insulator temperature. Therefore, the health condition of the equipment can be reflected by monitoring non-electrical parameters [1]. Temperature corresponds to a non-electrical quantity. Several types of monitoring devices exist for temperature information, including surface acoustic wave passive and wireless sensors, fiber Bragg grating sensors, Raman optical fiber distributed temperature sensors, and infrared image sensors [2,3,4,5]. When compared with other methods, the abnormal heating of power equipment can be effectively reflected by monitoring temperature via an infrared imager. Additionally, installation is simple and rapid due to its characteristic of non-contact temperature measurement. 

At present, most of the power equipment status diagnosis based on infrared images requires manual operation, which can only be conducted by experienced workers [6]. To automatically extract information including shape, position, size, and temperature from infrared images of power apparatus, target detection for abnormal parts must be performed via its infrared images [7]. Currently, target detection methods for normal images are mainly divided into two categories, namely those based on descriptors and based on artificial intelligence [8,9].

For the target detection method based on descriptors, researchers extracted features from various aspects, including gradient features, pattern features, shape features, and color features in recent years [10,11,12,13]. Zhao et al. [14] used the Binary Robust Invariant Scalable Keypoint (BRISK) and Vector of Locally Aggregated Descriptors (VLAD) to establish the shape features of the insulators. Zhai et al. [15] used the special color features of the insulator to determine the recognition area. Li et al. [16] proposed extracting shape features by Hough transform to identify corona discharge of transmission lines and insulators. Zhai et al. [17] proposed identifying insulators in aerial images by fusing their color and structural features. However, common problems exist in object detection only by descriptors such as poor robustness of the algorithm, complex structure of the features, and difficulty in multi-target matching in a single image.

Object detection algorithms based on artificial intelligence denote a new direction in the field of computer vision. Among them, the You Only Look Once (YOLO) series of networks corresponds to one-step convolutional neural networks [18,19], which are widely used for vehicle, pedestrian, and face recognition owing to their fast detection speed. To improve the recognition accuracy of the YOLO network, Joseph et al. [19] proposed You Only Look Once version 2 (YOLOv2) via various improvements based on the YOLO network. However, detection networks based on deep convolutional neural networks are mainly designed for horizontal or near-level general targets, and their rotation invariance is poor. Zhang et al. [20] added the feature layer of the rotation region to the traditional convolutional neural network. Yang et al. [21] proposed a detection model based on a multitasking rotational region convolutional neural network. Liu et al. [22] selected the optimal anchor box by using a two-level linear model and binary linear programming. However, the neural network algorithms are mainly aimed at high-resolution images. For infrared images with lower resolution, the recognition accuracy of the YOLOv2 network needs to be further improved.

In the study, the bushing in the infrared image of electrical equipment is used as the target detection object. Given the problems in the descriptors and neural networks, we propose a correction algorithm based on the YOLOv2 network. And this algorithm further improves the recognition accuracy based on YOLO2. First, in order to achieve the optimal recognition effect of the YOLOv2 network on a multi-angle object such as insulating bushing (IB), we propose using the standard Hough transform (SHT) to detect the approximate tilt angle of the object, and then calculating the optimal recognition angle of the recognized image by rotation. Second, the shape features of the IB are extracted by the SHT and the Gap statistic algorithm based on K-means clustering. Finally, the optimal bounding box (BB) is determined by the sliding window (SW).

The rest of this paper has been organized as follows. The structure and analysis of YOLOv2 is given in Section 2. The details of the proposed algorithm are described in Section 3. The result analysis is carried out in Section 4. Finally, conclusions and further work are outlined in Section 5.

## 2. Object Detection Based on YOLOv2 Network

The YOLOv2 network was proposed by Joseph et al. [19], which implemented many improvements when compared to the YOLO network [18]. The most significant improvement involves adding batch normalization to all convolutional layers to standardize the model and discard more redundant information. When compared with the YOLO network, YOLOv2 exhibits improved recognition accuracy and speed. However, the YOLOv2 and YOLO network are approximately similar in structure, and both belong to a one-step network [23].

### 2.1. Detection Process of the YOLOv2 Network

**Figure 1:**
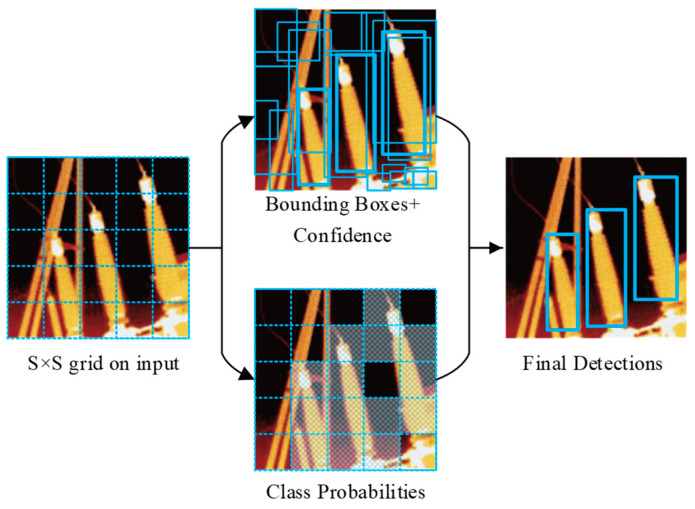
You Only Look Once version 2 (YOLOv2) network target detection process diagram.

The target detection process of YOLOv2 is as follows: First, the image is divided into S × S grids. Second, when the center pixel of the object ground truth (OGT) is in a grid, then the grid is responsible for detecting the object, and each grid predicts the information (*x*, *y*, *w*, *h*) and confidence of the BB. Specifically, *x*, *y*, *w*, and *h* represent the coordinates, width, and height of the BB, respectively. When multiple BBs detect the same target, YOLOv2 uses non-maximum suppression (NMS) algorithm to select the optimal BB as the final one [24]. The difference from the two-step network is that the YOLOv2 network synthesizes region prediction and classification prediction into a neural network model, thus its detection process is much faster than the two-step network [25,26]. The process is shown in Figure 1.

### 2.2. Defects in YOLOv2 Training Results

The YOLOv2 network is trained by the infrared image data set of the IB. In actual detection results, although the recognition rate is high, there are still the following problems:

(1) In multiple sets of images, the IBs with different tilt angles have poor recognition results.
(2) Some recognition results show a large deviation between the OGT and BB, and the Intersection-over-Union (IoU) is low, which affects the recognition accuracy.

## 3. Modified Algorithm for YOLOv2 Network

**Figure 2:**
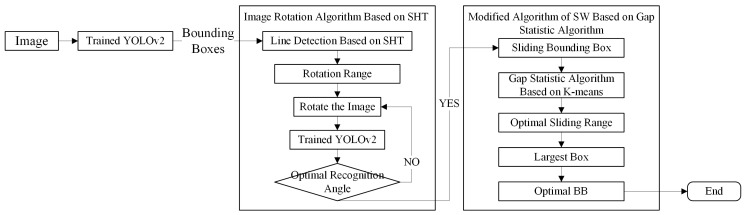
Our proposed algorithm flow chart.

Given that the rotation invariance of the YOLOv2 network is poor, the recognition effect of multi-angle IBs cannot meet expectations. It is also because the resolution and contrast of the infrared image is low, which affects the overall recognition effect of the YOLOv2 network. In view of the problems mentioned above, an image rotation algorithm based on the SHT is proposed such that the trained network can achieve the optimal recognition effect for different pictures. Furthermore, it is combined with the SHT and Gap statistic algorithm based on K-means clustering to extract the shape features of the IB. Finally, the optimal BB is determined by the SW. The specific process is shown in Figure 2.

### 3.1. Image Rotation Algorithm Based on Standard Hough Transform

In the infrared image data set, if the tilt angles of the same object are various, the recognition effect of the YOLOv2 network is also different. However, in practice, the inclination angle of the IBs are different. Therefore, if the trained YOLOv2 network is utilized to detect the infrared images of the IB, the problem of poor recognition accuracy may occur. Hence, it is necessary to rotate the recognized image to achieve the optimal network recognition effect.

#### 3.1.1. Feature Extraction Algorithm Based on Standard Hough Transform

When there are many identified objects in the infrared image, multiple corresponding BBs may be generated through the YOLOv2 network. The algorithm in this section uses SHT to detect straight lines in each BB that have been recognized by the YOLOv2 network and uses these lines as shape features. The following specific method is only for one BB.

**Figure 3:**
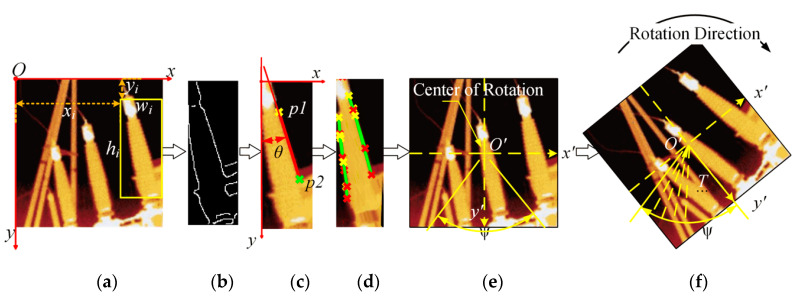
Schematic diagram for obtaining the optimal recognition angle; (**a**) recognition results of the YOLOv2 network; (**b**) extraction of image edges by Canny; (**c**) detection of straight line in the image by standard Hough transform (SHT); (**d**) the shape features of insulating bushing (IB); (**e**) determine rotation range ψ; (**f**) rotate the image.

The trained YOLO network is utilized to recognize the image, and BB {*x_i_*, *y_i_*, *w_i_*, *h_i_*} (*i* = 0, 1, …, *n*) is obtained finally, as shown in Figure 3a. Simultaneously, confidence *score_i_* is predicted.

Then, the edges within the BB are extracted by the Canny algorithm [27], as shown in Figure 3b.

We detect the segments in the edges by using the SHT. This algorithm utilizes the duality of points and lines to transform a point in the original space into a straight line in the parameter space. It turns the problem of line detection into a problem of finding the local maximum in the parameter space [14]. The algorithm is shown as follows:

*D* = *X* sin*θ + Y* sin*θ*, −90° < *θ* < 90° (1)

where *X* and *Y* are coordinates of any point in the detected area; *D* denotes the distance from the center of rotation (the center pixel *O’* of the image, as shown in Figure 3e) to the line in the direction perpendicular to the line; *θ* denotes the angle between the straight line and the y-axis, as shown in Figure 3c, and the figure shows the case where *θ* > 0°. Each point (*X*, *Y*) in the image is transformed into a sine curve in the parameter space (*D*, *θ*) by using Equation (1). If there is a straight line in the original input image, then the points on the straight line are converted to the parameter space, and the formed curves must intersect at one point, and thus the accumulated value of the point corresponds to a local maximum of the parameter space. A straight line in image space can be detected via detecting the local maximum and its corresponding parameters (*D*, *θ*), and the straight lines corresponding to the top 20% of all local maximum are selected as the recognition result because these straight lines are more salient in an image.

Thus, {*p1*, *p2*, *θ*, *D*} of the segment can be obtained via SHT, *p1* and *p2* are the coordinates of the starting point and the ending point on the straight line, respectively, as shown in Figure 3c. Because IB is a column, most of the identified segments are located at the edges of both sides. Thus, the shape features of IB can be extracted through the SHT, as shown in Figure 3d.

#### 3.1.2. Image Rotation Range

The approximate tilt angle of the identified objects in all BBs are calculated from the segments angle *θ* obtained above, and then the rotation range of image can be determined. The specific method is as follows:

We calculate the average of angles with respect to the identified segments in all BBs.

(2) $$Angle=\sum_{i=0}^{n}\sum_{j=1}^{m}\left(\theta_{ij}\right)/(m\times n)$$

where *Angle* denotes the overall tilt angle of the image. Additionally, *θ_ij_* denotes the angle of the detected segment *j* (*j* = 1, 2, …, *m*) in BB *i*, and *m* denotes the total number of detected straight lines in a BB.

We consider the clockwise direction as the positive direction, and the rotation range ψ is as follows:

(3) ψ=[−Angle, Angle]

Therefore, a fan-shaped rotation region is eventually formed, as shown in Figure 3e. During the rotation process, the entire rotation interval is equally divided with *O’* as the center of rotation to obtain *T* rotated images of one image, as shown in Figure 3f.

#### 3.1.3. Optimal Recognition Angle

In the *T* rotated images, we calculate the sum of the BBs’ confidence in each image. When the sum of the confidence reaches the maximum, the corresponding image is considered to reach the optimal angle for recognition. This is expressed as follows:

(4) $$Angle_{best}=\{Angle_{t}|\max(\sum_{i=1}^{n}score_{i})\}$$

where *Angle_best_* denotes the optimal recognition angle of the image, and *Angle_t_* denotes the *t*-th rotation angle of the image (*t* = 1,2, …, *T*). According to Equation (4), the image is rotated to the angle of *Angle_best_*, and then the new BB {*X_i_, Y_i_, W_i_, H_i_*} is determined via the YOLOv2 network.

### 3.2. Modified Algorithm of Sliding Window Based on Gap Statistic Algorithm

The currently trained YOLOv2 network can achieve the optimal recognition effect via the above modified algorithm, but some BBs are offset from the OGT, and thus room for improvement still exists.

**Figure 4:**
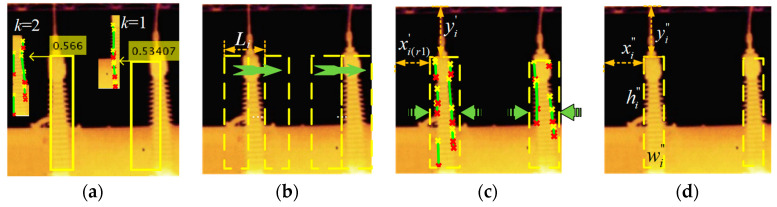
Schematic diagram of the sliding window (SW) correction process using the Gap statistic algorithm; (**a**) {*X_i_*, *Y_i_*, *W_i_*, *H_i_*} obtained under the optimal recognition angle; (**b**) translate the bounding boxes (BB); (**c**) largest box obtained by the SW algorithm; (**d**) determine the optimal BB.

Based on the algorithm in Section 3.1, after rotating the image to the optimal recognition angle, we proposed the SW algorithm to determine the optimal BB for target detection, and SW means to translate BB horizontally. First, the SHT is utilized to detect the straight line in each of translated BB and extract the shape features of the IB. Second, the position and angle of straight lines are used as cluster samples, and the optimal cluster number is calculated via the Gap statistic algorithm based on K-means clustering. Third, the number of clusters is utilized to reflect whether the current BB includes one side or two sides of IB. When the number of clusters is one, it means that the straight lines in BB are mainly distributed on one side. When the number of clusters is two, it means that they are distributed on both sides. Therefore, the area where the number of clusters corresponds to two during the BB translation is considered as the largest box. Finally, the optimal BB is determined by the largest box. The specific process is shown in Figure 4.

#### 3.2.1. Bounding Box Sliding Range

**Figure 5:**
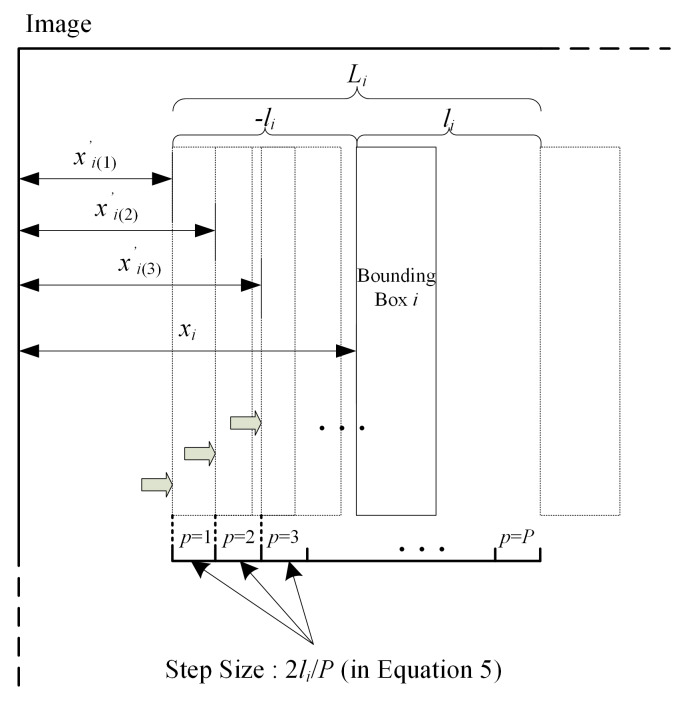
The translation process of the *i*-th BB.

We assume that the translation amount is *l_i_* = 0.5 × *W_i_*, where *l_i_* denotes the translation amount of the BB *i*. The BB is translated equidistantly in the range of *L_i_* = [−*l_i_*, *l_i_*]. Assuming that *L_i_* is equally divided into *P* intervals, then *P* translation distances are generated, and BB corresponding to *P* translation distances generates a total of P boxes. Their abscissas are shown in Equation (5) as follows:

(5) $$x_{i(p)}^{\prime }=x_{i}-l_{i}+p\times \frac{2l_{i}}{P}$$

where $x_{i(p)}^{\prime }$ denotes the abscissa of the *i*-th BB corresponding to the *p*-th translation distance in the translation range *L_i_*, and *P* defaults to 10 by experience. The moving process of BB in the sliding range is shown in Figure 4b and Figure 5.

#### 3.2.2. Gap Statistic Algorithm Based on K-Means

In Section 3.2.1, each BB will produce many boxes with different positions during the translation process. The SHT is utilized to perform straight line detection in each box $\left\{x_{i(p)}^{\prime },Y_{i},W_{i},H_{i}\right\}$, and the set {***θ*, *D***} of straight lines in each box is selected as the cluster sample. Simultaneously, to remove the deviation lines, the samples should satisfy −D(***θ***) ≤ *θ_j_ ≤* D(***θ***), and D (•) denotes the Variance. Based on K-means clustering, the optimal clustering number is determined by the Gap statistic algorithm [28], to determine whether the current translated BB contains one-side or two-sides of the IB. The specific algorithm is as follows.

The clustering sample is {*θ_j_*, *D_j_*} (*j* = 1, 2, …, *m*) in a BB, and they are clustered into *k* classes by K-means clustering algorithm. The clustering result is *C* = {*C_1_*, *C_2_*, …, *C_k_*}, and the sum of the sample distances *G_r_* in each class is as follows:

(6) $$G_{r}=\sum_{j,j^{\prime }\in C_{r}}d_{j,j^{\prime }},\;C_{r}\in C$$

(7) $$d_{j,j^{\prime }}=\sum \sqrt{{\left(\theta_{j}-\theta_{j^{\prime }}\right)}^{2}+{\left(D_{j}+D_{j^{\prime }}\right)}^{2}}$$

where *G_r_* denotes the sum of the distances about any two sample points in the class *r*, and $d_{j,j^{\prime }}$ denotes the Euclidean distance between the sample point *j* and the sample point *j^’^*.

(8) $$W(k)=\sum_{r=1}^{k}\frac{1}{2n_{r}}G_{r}$$

where *n_r_* denotes the total number of sample points in the class *C_r_*. We determine the optimal number of clusters via the Gap statistic algorithm.

(9) $$Gap(k)=min(E_{m}^{*}[log(W(k))]-log(W(k))|k=\{1,2\})$$

where $E_{m}^{*}$ denotes the Expectation of *m* samples under a given reference distribution, and *k* is 1 or 2. If *k* = 1, it implies that the current BB mainly includes one side of the IB; if *k* = 2, it implies that the current BB includes both sides of the IB. Therefore, we can utilize *k* to determine whether the BB contains a complete insulation bushing, as shown in Figure 4a.

The algorithm in this section is utilized to obtain the optimal clustering number of each BB in the *N* boxes generated by the translation, i.e., *K_i_* = {*k_1_*, *k_2_*, …, *k_r_*, …,*k_N_*} (*r* = 1, 2, …, *N*), and *K_i_* denotes the set of optimal clustering number for BB *i*.

#### 3.2.3. Optimal Sliding Range

In *K_i_*, if *k_r_* = 2 appears continuously, it implies that the corresponding BBs include both sides of the IB, and its translation range contains the main part of the IB. The optimal sliding range of the SW is determined by Equation (10) as follows:

(10) $$\left\{k_{r1},\ldots ,k_{r2}\}=\{K_{i}|\{\max\{long\{k_{r}=2\}\}\&(\sum_{r=1}^{N}k_{r}\neq N)\}\right\}$$

where *long*{•} denotes the continuous length of *k_r_* = 2 in *K_i_*. {*k*_*r*1_, …, *k*_*r*2_} (*k*_*r*1_, *k*_*r*2_ ∈ *K_i_*) denotes arrays of maximum continuous length when *k*_*r*1,…,*r*2_ = 2. Furthermore, *r*1 denotes the start index and *r*2 denotes the end index.

We map {*k*_*r*1_, …, *k*_*r*2_} to the translation range *L_i_* to obtain the position and width of the largest box.

(11) $$\left\{k_{r1},\ldots ,k_{r2}\}\Rightarrow \{x_{i(r1)}^{\prime },\ldots ,x_{i(r2)}^{\prime }\right\}$$

(12) $$w_{i}^{\prime }=w_{i}+(x_{i(r1)}^{\prime }-x_{i(r2)}^{\prime })$$

(13) $$y_{i}^{\prime }=Y_{i},\;h_{i}^{\prime }=H_{i}$$

where $x_{i(r1)}^{\prime }$ denotes the abscissa of the starting point in the optimal translation range, which is also the abscissa of the largest box, and $x_{i(r2)}^{\prime }$ denotes the abscissa of the ending point in the optimal translation range, *w_i_* and $h_{i}^{\prime }$ denote the width and height of largest box, respectively, and $y_{i}^{\prime }$ denotes the ordinate of the upper-left corner. We use Equations (11)–(13) to merge all the translated boxes of a BB in the optimal sliding range into the largest box, as shown in Figure 4c. The largest box is wider than the BB detected directly by YOLOv2, but it can better contain the identified target.

In addition to the above. When the elements in *K_i_* are all one, it indicates that in the process of BB translation, the shape features may not be discriminated due to the narrow width or the effect of background noise. At this time, {*X_i_, Y_i_, W_i_, H_i_*} is no longer modified.

#### 3.2.4. Optimal Bounding Box

The optimal BB is obtained by compressing the largest box horizontally. Straight line detection is performed on each of largest box through the SHT, and we obtain {*p*1, *p*2, *θ*, *D*} (−D(*θ*) ≤ *θ_j_ ≤* D(*θ*)).

(14) $$\left\{ \begin{array}{l} x_{i}^{\prime \prime }=\min(p1.x,p2.x)\\ w_{i}^{\prime \prime }=\max(p1.x,p2.x)-x_{i}^{\prime \prime }\\ y_{i}^{\prime \prime }=y_{i}^{\prime },\;h_{i}^{\prime \prime }=h_{i}^{\prime } \end{array}\right.$$

where $x_{i}^{\prime \prime }$ and $y_{i}^{\prime \prime }$ denote the abscissa and ordinate of the optimal BB, respectively. $w_{i}^{\prime \prime }$ and $h_{i}^{\prime \prime }$ denote the width and height, respectively. Specifically, $\left\{x_{i}^{\prime \prime },y_{i}^{\prime \prime },w_{i}^{\prime \prime },h_{i}^{\prime \prime }\right\}$ denotes the optimal BB as shown in Figure 4d.

### 3.3. Bounding Box Modified Algorithm Based on YOLOv2 Network

Bounding Box (BB) modified algorithm Based on YOLOv2 Network is showed in Algorithm 1.

**Algorithm 1:** BB modified algorithm based on YOLOv2 network**Input:** Image, prediction results of the YOLOv2 network {*x_i_*, *y_i_*, *w_i_*, *h_i_*} and *score_i_*
**Output:**
$\left\{x_{i}^{\prime \prime },y_{i}^{\prime \prime },w_{i}^{\prime \prime },h_{i}^{\prime \prime }\right\}$
**1:** Feature extraction of SHT: $\{x_{i},y_{i},w_{i},h_{i}\}\to \theta$**2:** Rotation range *ψ*: θ→Angle; Angle→ψ**3:** Best recognition angle algorithm: $\psi \to Angle_{best}$**4:** YOLOv2, $Angle_{best}\to \{X_{i},Y_{i},W_{i},H_{i}\}$**5:** Sliding position of BB: $\left\{X_{i},Y_{i},W_{i},H_{i}\}\to \{x_{i(p)}^{\prime },Y_{i},W_{i},H_{i}\right\}$**6:** SHT: $\left\{x_{i(p)}^{\prime },Y_{i},W_{i},H_{i}\}\to \{\theta_{j},D_{j}\right\}$**7:** Gap statistic algorithm based on K-means: $\{\theta_{j},D_{j}\}\to K_{i}$
**8: if**
$\sum_{r=1}^{N}k_{r}\neq N$
**9:**  $K_{i}\to \{x_{i(r1)}^{\prime },y_{i}^{\prime },w_{i}^{\prime },h_{i}^{\prime }\}$; $\left\{x_{i(r1)}^{\prime },y_{i}^{\prime },w_{i}^{\prime },h_{i}^{\prime }\}\to \{x_{i}^{\prime \prime },y_{i}^{\prime \prime },w_{i}^{\prime \prime },h_{i}^{\prime \prime }\right\}$
**10: return**
$\left\{x_{i}^{\prime \prime },y_{i}^{\prime \prime },w_{i}^{\prime \prime },h_{i}^{\prime \prime }\right\}$

**11: if**
$\sum_{r=1}^{N}k_{r}=N$
**12:**  $\left\{x_{i}^{\prime \prime },y_{i}^{\prime \prime },w_{i}^{\prime \prime },h_{i}^{\prime \prime }\}=\{X_{i},Y_{i},W_{i},H_{i}\right\}$
**13: return**
$\left\{x_{i}^{\prime \prime },y_{i}^{\prime \prime },w_{i}^{\prime \prime },h_{i}^{\prime \prime }\right\}$

**14: End**


## 4. Experiment and Result Analysis

In this experiment, we conducted contrast experiments on the infrared images of the power apparatus bushings to evaluate the effectiveness of our method. Test environment: CPU: Intel (R) Core (TM) i5-5200 CPU @ 2.20 GHz (4 CPUs); Memory: 8192 MB RAM; GPU: NVIDIA GTX 1050; Operating System: Windows 10; Matlab 2019b.

### 4.1. Experimental Data

We used an infrared camera (FLIR-A310) to acquire infrared images of IB. All pictures were taken from the front of IB according to the requirements of condition monitoring and included the main structure of IB [29]. The original sample contains 600 pictures. The main object in these images is IB, and there may be other objects in the background.

There are several ways to build a data set, and data augmentation avoids overfitting of the network and improves the effect of network recognition [30,31]. The data set can be enhanced via several methods. The specific data augmentation method is shown in Table 1.

**Figure 6:**
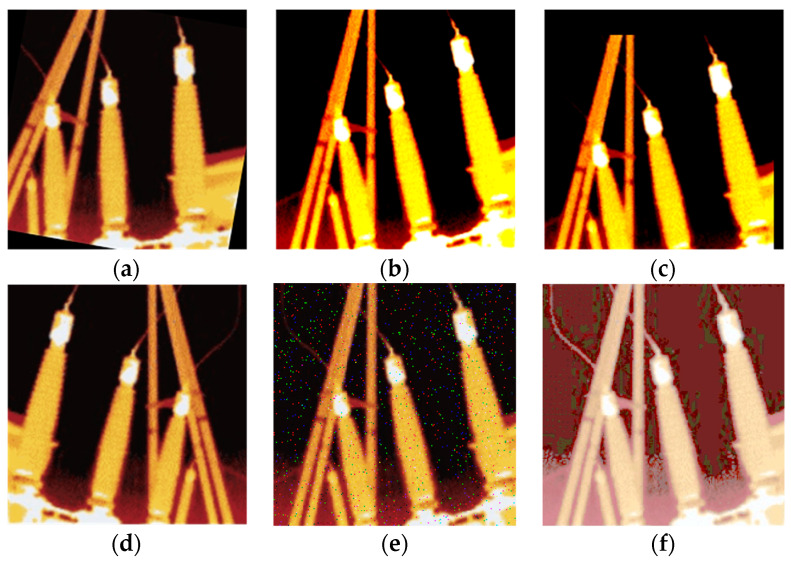
Schematic diagram of data augmentation: (**a**) rotating, (**b**) contrast changing, (**c**) translation, (**d**) mirroring, (**e**) salt and pepper noise, (**f**) histogram equalization.

The data augmentation effect of a few pictures is shown in Figure 6.

The final total data set contains 3000 pictures of 224 × 224 pixels. The insulation bushings were labeled based on the format of the VOC2007 data set, and these marked boxes are used as OGT [32]. Eighty percent of them are randomly divided into the training set and the rest of them are the test set.

### 4.2. Network and Parameters

We set the YOLOv2 detection network. First, Resnet-50 was used as the basic network, and activation_43 was selected as the feature extraction layer. This layer exhibits image feature coding capability and spatial resolution. Second, we deleted all the networks after the feature extraction layer and added detection subnets. The detection subnets are composed of a series of serially connected convolution layers, activation function Relu, and batch normalization layer. Finally, the YOLOv2 conversion layer and the YOLOv2 output layer were added to the detection subnet [33].

The anchor box is an important hyperparameter of the YOLOv2 network and should be determined through clustering. The anchor box is determined by the size of the object in the training set. The shape, size, and number of the anchor boxes affect the final recognition accuracy of the network. Based on the K-means++ algorithm [34], we used IoU as a distance metric to aggregate OGT with similar aspect ratios into one class and then generate anchor box estimates that are suitable for the dataset.

**Figure 7:**
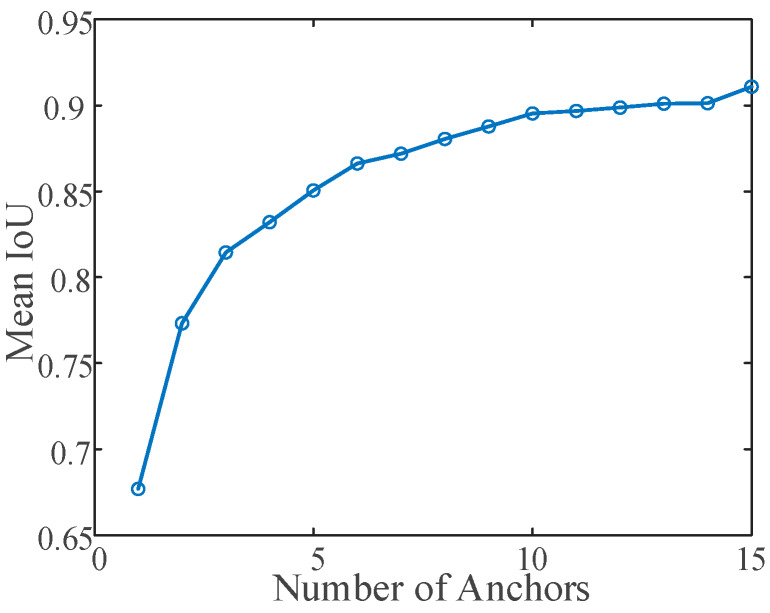
Number of anchor boxes and mean Intersection-over-Union (MIoU).

When the mean Intersection-over-Union (MIoU) exceeds 0.6, it indicates that a good overlap exists between the anchor box and OGT. A larger MIoU indicates a higher coincidence. Increases in the number of clusters can improve the MIoU although an excessive number of clusters increases the calculation cost and easily leads to overfitting. Hence, the number of clusters denotes a hyperparameter. As shown in Figure 7, when the number of clusters exceeds 4, the MIoU does not increase significantly. Therefore, the number of clusters selected in the experiment corresponds to 4.

The small batch stochastic gradient descent (SGD) method with a momentum factor was utilized to train the network, and the momentum factor corresponded to 0.9. The weight attenuation was set to 0.005 to prevent the model from overfitting. The batch size corresponded to 8. The regularization coefficient corresponded to 0.0005. The initial learning rate was set to 0.001.

### 4.3. Model Evaluation

In this experiment, Precision-Recall Curve (PRC), MIoU, and mean Average Precision (mAP) were used as evaluation indicators to evaluate the experimental results [35,36].

#### 4.3.1. Precision-Recall Curve

Specifically, PRC denotes a curve that intuitively reflects model performance. The precision is on the y-axis and the recall is on the x-axis. The formulas of precision and recall are as follows:

(15) $$\left\{ \begin{array}{l} precision=\frac{TP}{TP+FP}\\ TPR=\frac{TP}{TP+FN} \end{array}\right.$$

where *TPR* is recall, *TP* denotes a positive sample that is predicted by the model as positive, *FP* denotes a negative sample that is predicted by the model as positive, and *FN* denotes a positive sample that is predicted by the model as negative.

There exists a balance between precision and recall. In the ideal case, the PRC reaches the upper right corner, and the precision and recall both correspond to one, which denotes the optimal position for the curve. However, in practice, if the performance of a classifier is good, then the precision is maintained at a very high level while the recall increases [37].

#### 4.3.2. Mean Intersection-Over-Union

*IoU* is also an evaluation index for target detection, which denotes the overlap ratio between the *BB* and *OGT* [38]. Hence, it denotes the intersection of the *BB* and *OGT*. The increase of *IoU* represents the improvement of the model recognition effect. The *MIoU* denotes the mean of IoU about all BBs.

(16) $$IoU=\frac{BB\cap OGT}{BB\cup OGT}$$

*MIoU* = *mean*{*IoU*} (17)

#### 4.3.3. Mean Average Precision

Specifically, *AP* denotes the average precision of each class, and *mAP* denotes the mean *AP* of all classes [39]. This is expressed as follows:

(18) $$mAP=\frac{\sum AP}{N(Classes)}$$

where, *N(Classes)* denotes the number of classes. The target detection object in the paper is only IB, and thus *N(Classes)* = 1.

### 4.4. Comparison of Different Algorithms

To verify the performance of the algorithm proposed in this paper, the proposed model is compared with YOLO, YOLOv2, and YOLOv3, and in order to increase the reliability of the contrast experiments, the parameters of all algorithms were set according to the parameters in Section 4.2.

**Figure 8:**
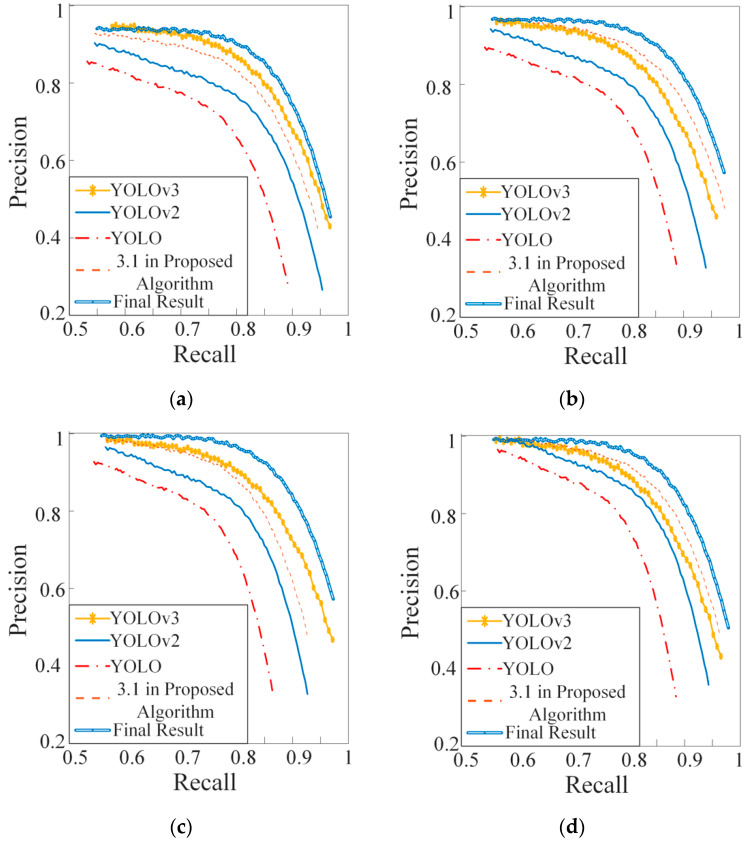
Precision-Recall Curve (PRC). “3.1 in Proposed Algorithm” represents the improvement of YOLOv2 only through “Image rotation algorithm based on SHT”; "Final Result" represents the result corresponding to the complete algorithm proposed in this paper. (**a**) Iteration 5000, (**b**) iteration 10,000, (**c**) iteration 15,000, (**d**) iteration 20,000.

As shown in Figure 8, the experiment results included PRCs of algorithms with different iterations. With the increase of iterations in all algorithms, the recognition accuracy reflected by the PRCs is continuously improved. We compared the curves corresponding to “YOLOv2”, “3.1 in Proposed Algorithm”, and “Final Result” in Figure 8, the image rotation algorithm based on the SHT (3.1 in Proposed Algorithm) leads to a significant improvement, and the SW improves the recognition accuracy again (Final Result). Thus, the proposed algorithm improves the target detection capability of the traditional YOLOv2 network. At the same time, the PRC in Figure 8 show that the proposed algorithm is also superior to YOLO and YOLOv3.

The data in Table 2 are mainly utilized to compare the YOLOv2 with the proposed algorithm. Compared with the recall of YOLOv2, the recall of “3.1” increases significantly although the MIoU does not increase significantly. This is because the algorithm in 3.1 only increases the number of correct detections and does not correct the offset of BB. "Final Result" represents the result corresponding to the complete algorithm proposed in this paper. Compared with the MIoU of 3.1, the MIoU of “Final Result” increases significantly in Table 2. This is because SW algorithm is mainly to reduce the deviation of BB, and the mAP of the proposed algorithm is higher than YOLOv2. Therefore, the algorithm in this paper improves the recall, mAP, and MIoU of YOLOv2.

The algorithms in Table 3 achieve the best training effect when the iteration is 20,000. Therefore, the data in Table 3 are the mAP of all algorithms when iteration is 20,000. Because the mAP of the proposed algorithm is the highest in Table 3, the data reflect that the recognition accuracy of the algorithm in this paper is the best.

**Figure 9:**
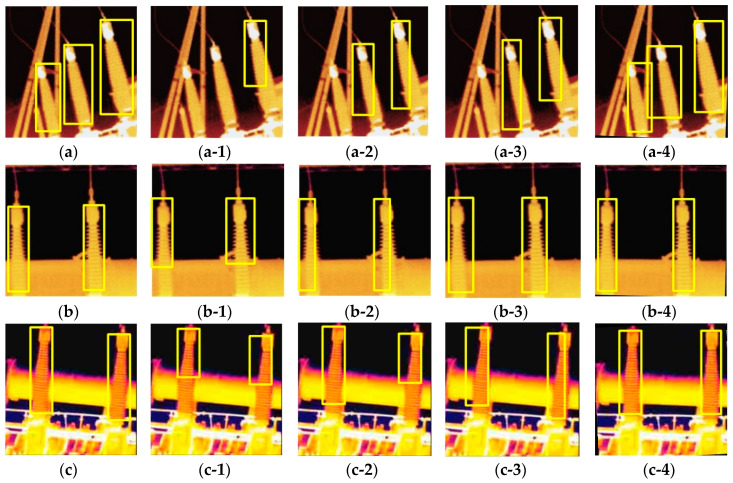
Recognition effect diagram of different algorithms. Object ground truth (OGT) as identification standard is manually marked. (**a-4**), (**b-4**), and (**c-4**) are marked by our algorithm. (**a**) OGT (**a-1**) YOLO, MIoU = 0.136 (**a-2**) YOLOv2, MIoU = 0.479 (**a-3**) YOLOv3, MIoU = 0.593 (**a-4**) MIoU = 0.724 (**b**) OGT (**b-1**) YOLO, MIoU = 0.391 (**b-2**) YOLOv2, MIoU = 0.694 (**b-3**) YOLOv3, MIoU = 0.792 (**b-4**) MIoU = 0.946 (**c**) OGT (**c-1**) YOLO, MIoU = 0.342 (**c-2**) YOLOv2, MIoU = 0.504 (**c-3**) YOLOv3, MIoU = 0.694 (**c-4**) MIoU = 0.894.

Figure 9 shows the actual recognition results of YOLO, YOLOv2, YOLOv3, and our algorithm. The resolution of the infrared images in Figure 9 is low, and the tilt angles of IBs in Figure 9a–c are different. We compared the recognition effects of YOLO, YOLOv2, and YOLOv3, and the YOLO algorithm has the worst recognition effect and the lowest MIoU. In Figure 9(a-2,a-3), both YOLOv2 and YOLOv3 miss a recognition target, and the BBs are too small in width to identify all parts of IBs. In Figure 9(a-4), we used the proposed algorithm to identify all IBs and correct all BBs with MIoU 0.724, and its recognition effect is better than YOLOv2 and YOLOv3. In Figure 9(b-2,b-3), although IBs are not inclined, recognition results show a deviation between the OGT and BB. In Figure 9(a-4,b-4,c-4), we utilized the proposed algorithm to rotate each image by a certain angle, the recognition effect is better than other algorithms.

To further verify the robustness of the proposed algorithm on multi-angle oblique objects, 600 test images were rotated at multiple angles according to Table 4, and we obtained the mAP of different algorithms when iteration is 20,000.

As seen in Table 4, the mAP of the proposed algorithm is the highest. Because the proposed algorithm can extract the shape features of IBs with different oblique angles and rotate images to the best recognition angle, it can fully utilize the recognition ability of the YOLOv2 network. Therefore, the proposed algorithm has higher robustness and is more suitable for identifying multi-angle oblique IBs.

## 5. Conclusions

The detection of electrical equipment via an infrared image is an important means for condition monitoring and fault diagnosis. Target detection can assist inspection robots and workers who are responsible for operation and maintenance. However, traditional techniques have limitations of target detection. Although algorithms based on descriptors are widely used for target detection in infrared and visible images, the recognition accuracy of such algorithms is low. The target detection algorithm based on a neural network is mainly used for high-resolution image. The resolution of the infrared image is relatively low, which seriously affects the recognition effect of a neural network. Thus, we tried to integrate them to overcome their drawbacks.

The proposed algorithm combines image processing methods with the YOLOv2 network. Given the poor rotation invariance of the network, the recognition effect is improved by rotating the image. Subsequently, the SHT and the Gap statistic algorithm based on K-means clustering are utilized to extract unique shape features of the IB, and BB is further modified by the SW. Finally, it is verified by three common target detection indicators. The results indicate that when compared with the traditional YOLOv2 network, the proposed algorithm significantly improves the recall, MIoU, and mAP, and compared with the recognition effect of YOLO and YOLOv3, the proposed algorithm fully reflects the advantages of recognition accuracy.

The proposed algorithm still has some operational limitations. When the recognized image is taken from the front, shape features can be effectively extracted. When the identified image cannot reflect the main shape of IB due to the shooting angle, the shape features cannot be extracted. However, for the condition monitoring and fault diagnosis of IB, the infrared image must reflect the condition of the entire IB so that workers can make an effective judgment. So, the algorithm in this paper has certain applicability.

The study fully considers the shape features of the IB in the infrared image, and the idea of correcting BB by the SW is useful to identify other types of electrical equipment in infrared images, such as busbars, voltage transformers, and circuit breakers.

## Figures and Tables

**Table 1:** Data augmentation.

Method	Rotating	Contrast Changing	Salt & Pepper
Amount	600	300	300
**Method**	**Translation**	**Mirroring**	**Histogram Equalization**
Amount	300	600	300

**Table 2:** Recall and MIoU.

Algorithm	YOLOv2	3.1 *	Final Result **
Iteration	5000		
Recall	0.6149	0.77	0.77
MIoU	0.4703	0.5091	0.6174
mAP	54.14%	70.62%	74.49%
Iteration	10,000		
Recall	0.7489	0.89	0.89
MIoU	0.5402	0.5943	0.6892
mAP	68.55%	82.43%	86.45%
Iteration	15,000		
Recall	0.8375	0.95	0.95
MIoU	0.6032	0.6666	0.7581
mAP	71.03%	93.11%	96.23%
Iteration	20,000		
Recall	0.8154	0.93	0.93
MIoU	0.6145	0.6759	0.7608
mAP	71.85%	93.33%	97.33%

* “3.1” is the improvement of YOLOv2 only through “image rotation algorithm based on SHT” in the proposed algorithm. ** “Final Result” is the result corresponding to the complete algorithm proposed in this paper.

**Table 3:** Mean Average Precision (mAP).

Algorithm	YOLO	YOLOv2	YOLOv3	Proposed Algorithm
mAP	58.85%	71.85%	91.23%	97.33%

**Table 4:** mAP at different rotation angles.

Rotation Angle	YOLO	YOLOv2	YOLOv3	Proposed Algorithm
−30°	30.48%	60.26%	71.42%	89.47%
−20°	49.43%	67.71%	86.74%	94.94%
−10°	55.52%	71.81%	89.49%	97.33%
10°	54.17%	73.04%	87.42%	96.36%
20°	50.92%	70.78%	82.38%	93.43%
30°	29.43%	61.41%	73.26%	90.41%
